# Self-assembled HCV core virus-like particles targeted and inhibited tumor cell migration and invasion

**DOI:** 10.1186/1556-276X-8-401

**Published:** 2013-09-27

**Authors:** Xiang Li, Xuehe Xu, Aihui Jin, Qunying Jia, Huaibin Zhou, Shuai Kang, Yongliang Lou, Jimin Gao, Jianxin Lu

**Affiliations:** 1 Zhejiang Provincial Key Laboratory of Medical Genetics, Wenzhou Medical University, Wenzhou, Zhejiang 325035, People’s Republic of China; 2 Zhejiang Provincial Key Lab for Technology and Application of Model Organisms, Wenzhou Medical University, Wenzhou, Zhejiang 325035, People’s Republic of China; 3 Nanfang Hospital, Southern Medical University, Guangzhou, Guangdong 510515, People’s Republic of China; 4 Clinical Laboratory of Ningbo Yinzhou Second Hospital, Ningbo, Zhejiang 315100, People’s Republic of China

**Keywords:** HCV core, Virus-like particles, VLPs, Tumor specificity, Migration, Invasion

## Abstract

We used a baculovirus expression system to express fusion proteins of HCV core, RGD (Arg-Gly-Asp) peptide, and IFN-α2a fragments in Sf9 cells. Western blotting and electron microscopy demonstrate that HCV core, peptides RGD, and IFN-α2a fusion proteins assemble into 30 to 40 nm nano-particles (virus-like particles, VLPs). Xenograft assays show that VLPs greatly reduced tumor volume and weight with regard to a nontreated xenograft. Migration and invasion results show that VLPs can inhibit the migration and invasion of the breast cancer cells MDA-MB231. This study will provide theoretical and experimental basis for the establishment of safe and effective tumor-targeted drug delivery systems and clinical application of VLPs carrying cell interacting cargo.

## Background

According to the World Health Organization (WHO), cancer is one of the leading causes of death worldwide (http://www.who.int/mediacentre/factsheets/fs297/en/index.html). Cancer control has therefore become a global health strategic focus. Treatment of malignant tumors traditionally involves a combination of surgery, radiation therapy, and chemotherapy. Surgery and radiation therapy are effective in addressing the local tumor; chemotherapy, however, carries severe toxicity due to lipid solubility and high therapeutic doses required for most cancers (>70%) [1]. With these therapeutic limitations, combination therapy has received close attention in the recent years. The addition of interferon (IFN) has become one of the most common additions to combination therapies.

In 1957, Isaacs and Lindenman discovered a secreted factor that actively interferes with and inhibits viral replication in influenza virus-infected chick embryo cells. They named the secreted factor interferon (IFN) and further classified the compound as either type I or II [2]. IFN conveys resistance to virus infection, inhibits tumor cell growth, and modulates the immune response of the organism. With such broad activity, IFN has become one of the most actively explored topics of immunology, genetics, virology, oncology, and molecular biology research [3]. Therefore, the development of cancer treatment programs aimed at tumor-specific molecular targets has become a focus of intense interest and research. Integrins are a family of cell adhesion receptors [4]. These receptors are heterodimeric transmembrane (TM) proteins containing two non-covalently associated α and β subunits. Integrins transmit bidirectional signals across the plasma membrane and regulate many biological functions, including cell differentiation, migration, growth, and survival. Integrins also play an important role in tumor invasion and metastasis [5,6]. Studies have shown that α_v_β_3_ is highly expressed not only on the cell surface of osteosarcoma, neuroblastoma, lung cancer, breast cancer, prostate cancer, bladder cancer, glioblastoma, invasive melanoma, and other solid tumors but also on neovascular endothelial cells of all tumor tissue [7-9]. Studies have demonstrated that RGD peptide (arginine-glycine-aspartic) can specifically bind and inhibit the activity of α_v_β_3_ integrin [10-12]. Thus, RGD is not only effective as a drug for the treatment of tumors but can also be effective in the targeting of tumor-associated molecules.

Nano-particles can provide tremendous advantages in drug and gene therapy [13]. A number of artificial polymers have been investigated extensively to formulate ideal, biodegradable nano-drug delivery carriers, such as polylactide, poly-L-lactic acid (PLLA), polycaprolactone (PCL), and poly(lactide-co-glycolide) (PLGA) [14]. Our pre-experiment research shows that HCV core protein can form HCV virus particles via baculovirus expression system. Virus-like particles (VLPs) are free of the virus genome and cannot cause infection. VLPs are the same size as nano-particles and appropriate as drug and gene therapy vectors [15-17].

In this study, we expressed HCV core, RGD peptide, and IFN-α2a fusion proteins by baculovirus expression system. We then have examined the specificity of the fusion protein binding to tumor cells and analyzed the effect of these fusion proteins on tumor cell migration and invasion. We further observed the function of these fusion proteins in a tumor xenograft mouse model. This study provides theoretical and experimental basis for the establishment of safe and effective tumor-targeted drug delivery systems and clinical application of VLPs.

## Methods

### Cell lines and viruses

*Spodoptera frugiperda* IPLB-Sf21-AE colonial isolate 9 (Sf9) cells were cultured at 27°C in Grace’s medium (Invitrogen, Carlsbad, CA, USA) with a supplement of 10% fetal bovine serum (FBS) (Invitrogen). MDA-MB231 human breast cancer cells, HCT116 human colon cancer cells, and 293 T human embryonic kidney cells were cultured in Dulbecco’s modified Eagle’s medium (DMEM) supplemented with 10% fetal bovine serum, 100 U/ml penicillin G, and 100 μg/ml streptomycin, at 37°C under 5% CO_2_, provided by Wuhan Institute of Virology, Chinese Academy of Sciences, China Center for Type Culture Collection (CCTCC, Wuhan, China).

### Reagents

Restriction endonuclease enzyme BamHI, EcoRI, SalI, nucleic acid molecular weight marker, DNA polymerase Pfu, DNA Marker, Gel extraction kit, and T4 DNA ligase were from TaKaRa (Shiga, Japan). Reverse transcriptase polymerase chain reaction (RT-PCR) and RNA extraction kits were purchased from Life Technologies Corporation (Grand Island, NY, USA). HCV core antibody was purchased from Shenzhen Jingmei Biotechnology Company (Shenzhen, China). Growth factor reduced Matrigel was purchased from BD Bioscience (San Jose, CA, USA). Ni-NTA Agarose (25 ml) was purchased from QIAGEN (Germantown, MD, USA). PureLink RNA kit and cDNA SuperScript First Strand Synthesis kit were from TaKaRa. Lipofectamine 2000 was purchased from Life Technologies Corporation. HRP-conjugated goat anti-rabbit secondary antibody was obtained from Abcam (Cambridge, MA, USA). West Pico ECL reagent was from Pierce (Rockford, IL, USA). Dulbecco’s modified Eagle’s medium (DMEM) and fetal bovine serum were purchased from Gibco (Grand Island, NY, USA). Penicillin G and 100 μg/ml streptomycin were purchased from Shanghai Biotechnology Company (Shanghai, China). DNA primers were synthesized by Shanghai Sangon Biotechnology Company (Shanghai, China).

### Methods

#### Expression and purification of RGD-IFN-α2a-core fusion protein

**Figure 1:**
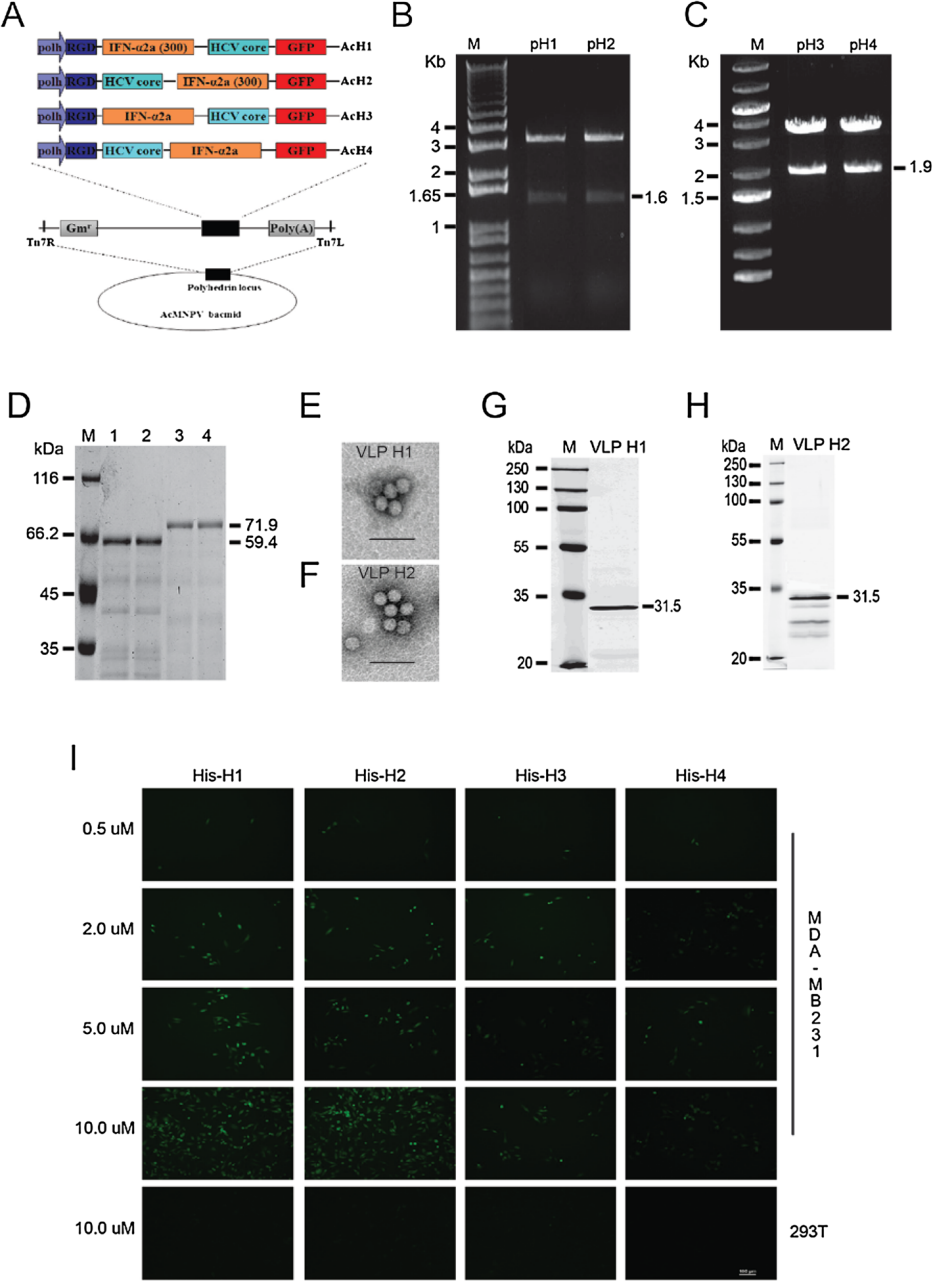
**RGD-core-IFN-α2a fusion proteins bind breast cancer cells MDA-MB231 in vitro. (A)** Recombinant bacmid constructs, showing the strategy for insertion of the gene cassettes into the polyhedrin locus of the AcMNPV bacmid. RGD-HCV core was fused with IFN-α2a. Both cassettes depicted were inserted into the attb site (indicated by the right and left insertion sites, Tn7R and Tn7L) in the polyhedrin locus by Tn-based transposition and generated the recombinant Bacmid: AcH1, AcH2, AcH3, and AcH4. **(B)** Identification of pH1 and pH2. M: 1Kb Plus DNA ladder; pH1 and pH2 samples were digested by BamHI and EcoRI. **(C)** Identification of pH3 and pH4. M: O’Gene Ruler 1Kb DNA ladder; pH3 and pH4 samples were digested by BamHI and EcoRI. **(D)** Purification of RGD-core-IFN-α2a fusion protein. M: protein marker; 1: His-H1; 2: His-H2; 3: His-H3; 4: His-H4. The recombinant bacmids AcH1, AcH2, AcH3, and AcH4 were introduced by transfection into Sf9 cells to produce the recombinant proteins His-H1, His-H2, His-H3, and His-H4. The fusion proteins were purified from the supernatants of cell lysates using Ni-NTA affinity resin. **(E**, **G)** Electron micrograph images and Western blotting result of VLP H1. Purified VLPs were attached onto a carbon-coated grid for 5 min at room temperature. The grid was rinsed with distilled water and stained with 1% phosphotungstic acid for 3 min before air drying on filter paper. The specimens were viewed using a Tecnai G2 transmission electron microscopy at 75 keV. For Western blot, 10 μg purified VLPs were separated by SDS-PAGE electrophoresis and subjected to Western blot assay. **(F**, **H)** Electron micrograph images and Western blotting result of VLP H2. **(I)** RGD-core-IFN-α2a fusion protein bind with breast cancer cells MDA-MB231. Then, 0.2, 0.5, 2, 5, and 10 μM fusion proteins His-H1, His-H2, His-H3, and His-H4 were co-incubated with MDA-MB231 at 37° under 5% CO_2_. After 2 h, the cells were washed three times with PBS, and green fluorescence was observed under the fluorescence microscope. Scale bar = 100 μm.

The complete EGFP gene was amplified by PCR using the pFastBacDual -EGFP as a template with the primers: 5’-TAGAATTCATGGTGA GCAAGGGC GAGGAG-3’/ 5’-ACGCGTCGACTTACTTGTACAGCTCGTC-3 and was cloned into the SalI and EcoRI sites of pFastBac HTb to produce pFastBac HTb-EGFP. RGD-IFN-α2a (300)-core (the PCR product length of IFN-α2a is 300 bp), RGD-core-IFN-α2a (300), RGD-IFN-α2a-core, and RGD-core-IFN-α2a fragments were amplified using pMD-RGD-IFN-α2a (300)-core, pMD-RGD-core-IFN-α2a (300), pMD-RGD-IFN-α2a-core, pMD-RGD-core-IFN-α2a as templates and 5’-TAGGATCCATGGTCGTGGCGATTGT-3’ / 5’-TAGAATTCGGCTGAAGCGGGCACAGT-3’ (RGD-IFN-α2a (300)-core /RGD-IFN-α2a-core); 5’-TAGGATCCATGT GTCGTGG CGATTGT-3’/ 5’-CGCGAATTCTTCCTTACTTCTTAAACTTTCTTG-3’ (RGD-core-IFN-α2a (300)); 5’-TAGGATCCATGTGTCGTGGCGATTGT-3’ / 5’-CCGGAATTCGAGTTCAGTGTAGAATTTGT-3’ (RGD-core-IFN-α2a) and subcloned into the pFastBacHTb-EGFP via BamH1/EcoRI sites and produced pFastBacHTb-EGFP -RGD-IFN-α2a (300)-core (pH1), pFastcHTb-EGFP-RGD-core-IFN-α2a (300) (pH2), pFastBacHTb-EGFP-RGD-IFN-α2a-core (pH3), and pFastBacHTb-EGFP-RGD-Core-IFN-α2a (pH4). All plasmids were sequenced by Beijing Genomics Institute. The four plasmids (pH1, pH2, pH3, and pH4) mediated the insertion of genes into the AcBacmid by Tn7-mediated transposition to generate AcH1, AcH2, AcH3, and AcH4 bacmids, respectively (Figure 1A). These recombinant bacmids were confirmed by PCR and were then introduced by transfection into Sf9 cells to produce the recombinant proteins His-H1, His-H2, His-H3, and His-H4. These four fusion proteins were purified by affinity chromatography using Ni-NTA agarose, according to according to the manufacturer’s directions (Qiagen, Carlsbad, CA, USA).

### Binding specificity assay

MDA-MB231 human breast cancer cells were cultured in DMEM supplemented with 10% fetal bovine serum, 100 U/ml penicillin G, and 100 μg/ml streptomycin, at 37°C under 5% CO_2_. Then, 0.2, 0.5, 2, 5, and 10 μM fusion proteins His-H1, His-H2, His-H3, and His-H4 were co-incubated with MDA-MB231 at 37°C under 5% CO_2_. After 2 h, the cells were washed three times with PBS, and green fluorescence was observed under the fluorescence microscope.

### Construction of recombinant baculovirus

pH1, pH2, pH3, and pH4 were digested by BamHI/EcoRI and were subcloned into pFastBac dual vector (pFBD) that had been pre-treated with BamHI/EcoRI and produced pFBD-H1, pFBD-H2, pFBD-H3, and pFBD-H4. The four donor plasmids (pFBD-H1, pFBD-H2, pFBD-H3, and pFBD-H4) mediated the insertion of genes into the AcBacmid by Tn7-mediated transposition to generate AcFBD-H1, AcFBD-H2, AcFBD-H3, and AcFBD-H4 bacmids, respectively. These recombinant bacmids were confirmed by PCR and then were introduced by transfection into Sf9 cells to produce the recombinant viruses vAcH1, vAcH2, vAcH3, and vAcH4.

### Real-time Q-PCR and Western blotting

Total RNA was extracted from cells with PureLink RNA kit (Life Technologies Corporation). cDNA was synthesized with SuperScript First Strand Synthesis kit (Invitrogen, Carlsbad, CA, USA) with 0.5 to 1.0 μg RNA according to the manufacturer’s instructions. Quantitative RT-PCR reactions were carried out using SYBR Green PCR master mix reagents on an ABI 7500 Fast Real-Time PCR System (Applied Biosystems, Foster City, CA, USA). The relative quantification of gene expression for each sample was analyzed by the Δ*C*_t_ method. The following primers were used to amplify HCV core: 5’- GCC CAC AGG ACG TCA AGT −3’ and 5’- CGC AAC CCT CAT TGC CAT −3’; 18S rRNA: 5’-ACC TGG TTG ATC CTG CCA GT-3’ and 5’-CTG ACC GGG TTG GTT TTG AT-3’.

Cells were harvested at 72 h post-infection (hpi) and lysed in SDS-PAGE loading buffer. Twenty micrograms of total protein was separated on a 12% sodium dodecyl sulfate polyacrylamide gel (SDS-PAGE) by electrophoresis and subjected to Western blot assay. The proteins were transferred to a membrane that was blocked in 5% milk 1 h at room temperature and incubated with the anti-HCV core monoclonal antibody (Jingmei Biotechnology Company; 1:1000 diluted in 5% milk) overnight at 4°C, followed by extensively washing with TBST (50 mM Tris–HCl, pH = 7.4, 150 mM NaCl, 0.1% Tween 20). The membrane was then incubated with the HRP-conjugated goat anti-rabbit secondary antibody (Abcam) for 1 h at room temperature before being developed with West Pico ECL reagent (Pierce).

### Purification and identification of virus-like particles

Sf9 cells were infected with BV (Budded Virus) of recombinant baculovirus at an MOI of 0.1. After 3 days, the cell culture supernatant was collected and clarified at 2,000 × *g* for 10 min at 4°C. The supernatant containing the BV was passed through a 0.45-mm pore-size filter. The filtrate was pelleted through a 25% (wt/wt) sucrose cushion in 0.1× Tris-buffered EDTA (TE) (TE: 10 mM Tris/HCl, pH 7.5, and 1.0 mM EDTA) at 100,000 × *g* for 90 min at 4°C, and resuspended in 0.1× TE. For negative staining, purified VLPs were attached onto a carbon-coated grid for 5 min at room temperature. The grid was rinsed with distilled water and stained with 1% phosphotungstic acid for 3 min before air drying on a filter paper. The specimens were visualized using a Tecnai G2 transmission electron microscope (FEI, Hillsboro, OR, USA) at 75 KeV.

### Xenografts and animal experiments

Animal experiments were performed following a protocol approved by the Institutional Animal Committee of Wenzhou Medical College. Thirty female nude mice (5 to 6 weeks old) were injected subcutaneously with 1 × 10^6^ MDA-MB231 breast cancer cells into the left and right mammary glands of each animal. Tumor size was measured daily or every other day with calipers, and tumor volumes were calculated using the formula: Volume = (width)^2^ × length/2. Once tumor volumes reached 250 mm^3^, animals were randomized into three groups (*n* = 5 animals/group): control, VLP H1, and VLP H2 (1 mg/kg body weight i.p. daily) were injected intraperitoneally (i.p.) into the mice. Mice were monitored daily for signs of toxicity, and body weight and tumor diameters were measured three times per week. Mice were euthanized 3 weeks later, and tumors were weighed.

### Cell migration assays

MDA-MB231 human breast cancer cells were treated with trypsin and resuspended in DMEM medium containing 1% FBS and 10 ng/ml EGF and 10 μM VLP H1 or VLP H2, plated at low densities on glass-bottomed dishes (MatTek, Ashland, MA, USA) coated with 5 μg/ml fibronectin and cultured for 3 h in a CO_2_ incubator. Cell motility was measured with a Nikon Biostation IMQ (Nikon Instruments Inc., Melville, NY, USA). Cell migration was tracked for 6 h; images were recorded every 10 min. The movement of individual cells was analyzed with NIS-Elements AR (Nikon).

### Invasion assays

One hundred microliters of Matrigel (1:30 dilution in serum-free DMEM medium) was added to each Transwell polycarbonate filter (6 mm in diameter, 8 μm in pore size, Costar, Washington, DC, USA) and incubated with the filters at 37°C for 6 h. Breast cancer cells MDA-MB231 were trypsinized and washed three times with DMEM containing 1% FBS. The cells were resuspended in DMEM containing 1% FBS at a density of 5 × 10^5^ cells per milliliter. The cell suspensions (100 μl) were seeded into the upper chambers, and 600 μl of DMEM medium containing 10% FBS and 10 μM VLP H1 or VLP H2 was added to the lower chambers. The cells were allowed to invade for 12 h in a CO_2_ incubator, fixed, stained, and quantitated as described previously [18].

## Results

### Expression and purification of fusion proteins

RGD-IFN-α2a (300)-core, RGD- core-IFN-α2a(300), RGD-IFN-α2a-core, and RGD-core-IFN-α2a fragments were amplified using pMD-RGD-IFN-α2a (300)-core, pMD-RGD- core-IFN-α2a(300), pMD-RGD-IFN-α2a-core, and pMD-RGD-core-IFN-α2a as templates and subcloned into the pFastBacHTb-EGFP via BamH1/EcoRI sites and produced pFastBacHTb-RGD-IFN-α2a (300)-core (pH1), pFastBacHTb-RGD-core-IFN-α2a(300) (pH2), pFastBacHTb-RGD-IFN-α2a-core (pH3), and pFastBacHT b-RGD-core-IFN-α2a (pH4). The expression vectors pH1, pH2, pH3, and pH4 were confirmed on an agarose gel after double digestions with BamHI and EcoRI (Figure 1B,C) and further confirmed by DNA sequencing. Finally, the successfully constructed expression vectors pH1, pH2, pH3, and pH4 mediated the insertion of genes into the AcBacmid by Tn7-mediated transposition to generate AcH1, AcH2, AcH3, and AcH4 bacmids, respectively (Figure 1A). These recombinant bacmids were introduced by transfection into Sf9 cells to produce the recombinant proteins His-H1, His-H2, His-H3, and His-H4. The fusion proteins were purified from the supernatants of cell lysates using Ni-NTA affinity resin under native conditions. Intense bands corresponding to the molecular weights of the expected proteins are shown: 59.4 kDa for His-H1 and His-H2; 71.9 kDa for His-H3 and His-H4; the concentration of His-H1 and His-H2 is higher than the His-H3 and His-H4 (Figure 1D).

### Identification of virus-like particles

To identify the impurities in the VLP H1 and VLP H2 preparation after crude purification with successive sucrose gradients, various analyses were performed. First, confirmation of protein identity in the VLP preparation was performed by immunoblotting using HCV core-specific monoclonal antibodies (Figure 1G,H). In addition, EM analysis of the protein was performed and revealed spherical VLPs of 30 to 40 nm in size (Figure 1E,F).

### RGD- core-IFN-α2a fusion protein specifically binds with cancer cell line

RGD (arginine-glycine-aspartic acid) can specifically bind with α_v_β_3_ integrin, which is highly expressed on the cancer cell surface. The recombinant RGD- core-IFN-α2a protein was expressed and purified in Sf9. As expected, the recombinant RGD- core-IFN-α2a can specifically bind breast cancer cells MDA-MB231 and colon cancer cells HCT116 (data not shown) but do not bind normal cells such as normal human embryonic kidney cell 293 T. The binding activities of His-H1 and His-H2 are stronger than the activity of His-H3 and His-H4 (Figure 1I). The binding activity with MDA-MB231 increased with fusion protein concentration (from 0.5 to 10 μM). When the protein concentration reached 10 μM, the binding activity was found to be at max capacity (Figure 1I).

### Real-time Q-PCR and translational analysis

**Figure 2:**
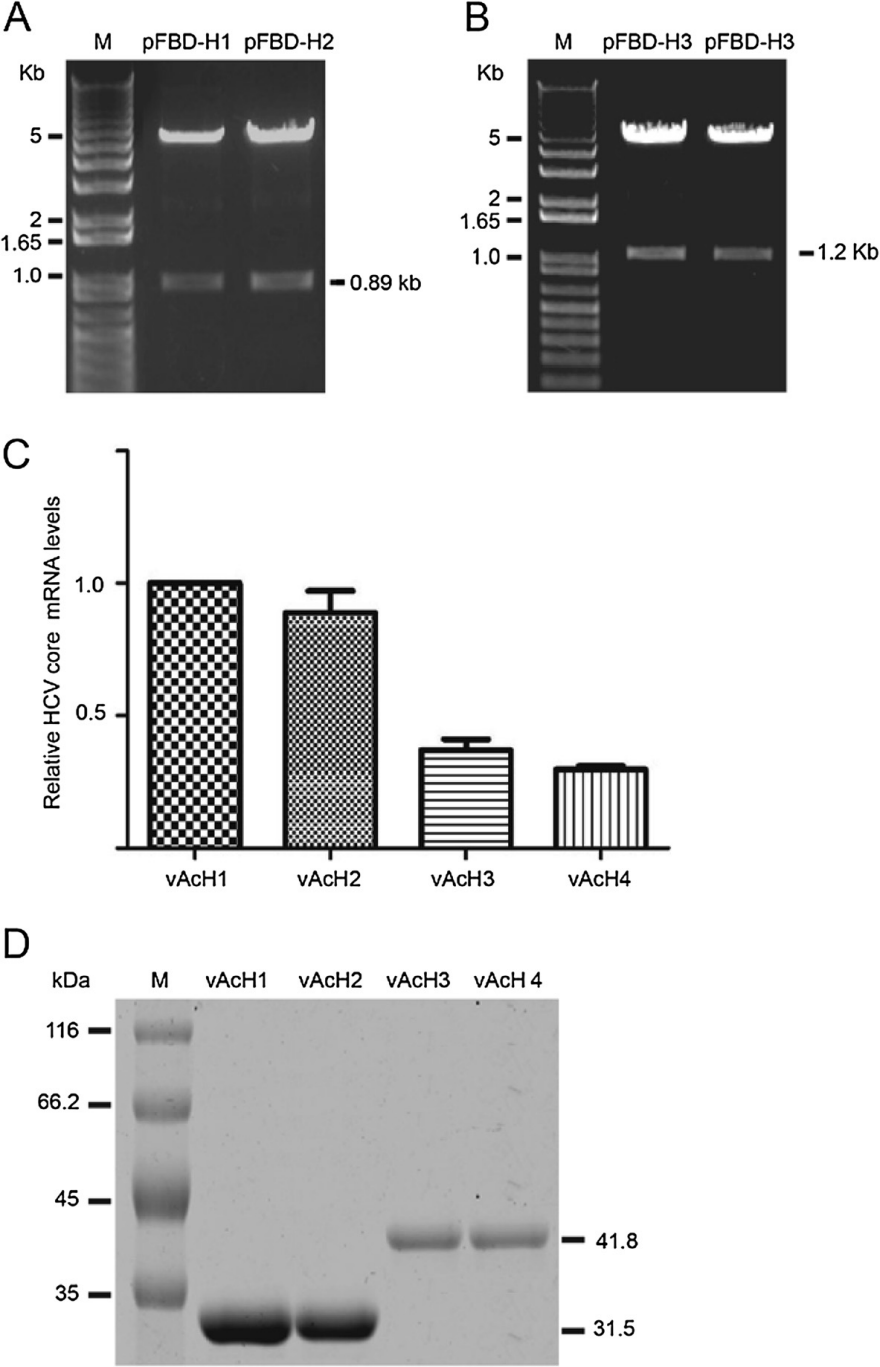
**Transcription and expression of HCV core-IFN-α2a recombinant viruses. (A)** Identification of pFBD-H1 and pFBD-H2. M: 1Kb Plus DNA ladder; pFBD-H1 and pFBD-H2 samples were digested by BamHI and EcoRI. **(B)** Identification of pFBD-H3 and pFBD-H4. M: 1Kb Plus DNA ladder; pFBD-H3 and pFBD-H4 samples were digested by BamHI and EcoRI. **(C)** RT-PCR results of HCV core gene in recombination viruses infect cells. Total RNA was isolated from Sf9 infected with vAcH1, vAcH2, vAcH3, or vAcH4. cDNA was synthesized with SuperScript First Strand Synthesis kit (Invitrogen) with 0.5 to 1.0 μg RNA according to the manufacturer’s instructions. Quantitative RT-PCR reactions were carried out using SYBR Green PCR master mix reagents on an ABI 7500 Fast Real-Time PCR System (Applied Biosystems). **(D)** Expression of HCV core-IFN-α2a fusion protein in recombinant virus infected cells. M: protein marker. Cells were harvested at 72 h post-infection (hpi) and lysed in SDS-PAGE loading buffer. Twenty micrograms of total protein was separated by SDS-PAGE and subjected to Western blot assay.

Transcription of RGD- core-IFN-α2a was examined by RT-PCR, using total RNA isolated from Sf9 cells infected with the recombinant virus vAcH1, vAcH2, vAcH3, and vAcH4. The transcriptional levels of vAcH1 and vAcH2 are higher than the vAcH3 and vAcH4 (Figure 2C). At the same time, the Western blotting results show that RGD-core-IFN-α2a expression levels in vAcH1 and vAcH2 are higher than levels from vAcH3 and vAcH4 (Figure 2D). From the results of binding, transcription, and translation analysis, we concluded that vAcH1 and vAcH2 are more effective on cancer cells. We then used vAcH1 and vAcH2 to analyze the VLP functions.

### vAcH1 and vAcH2 inhibit breast cancer cells MDA-MD-231 migration and invasion

**Figure 3:**
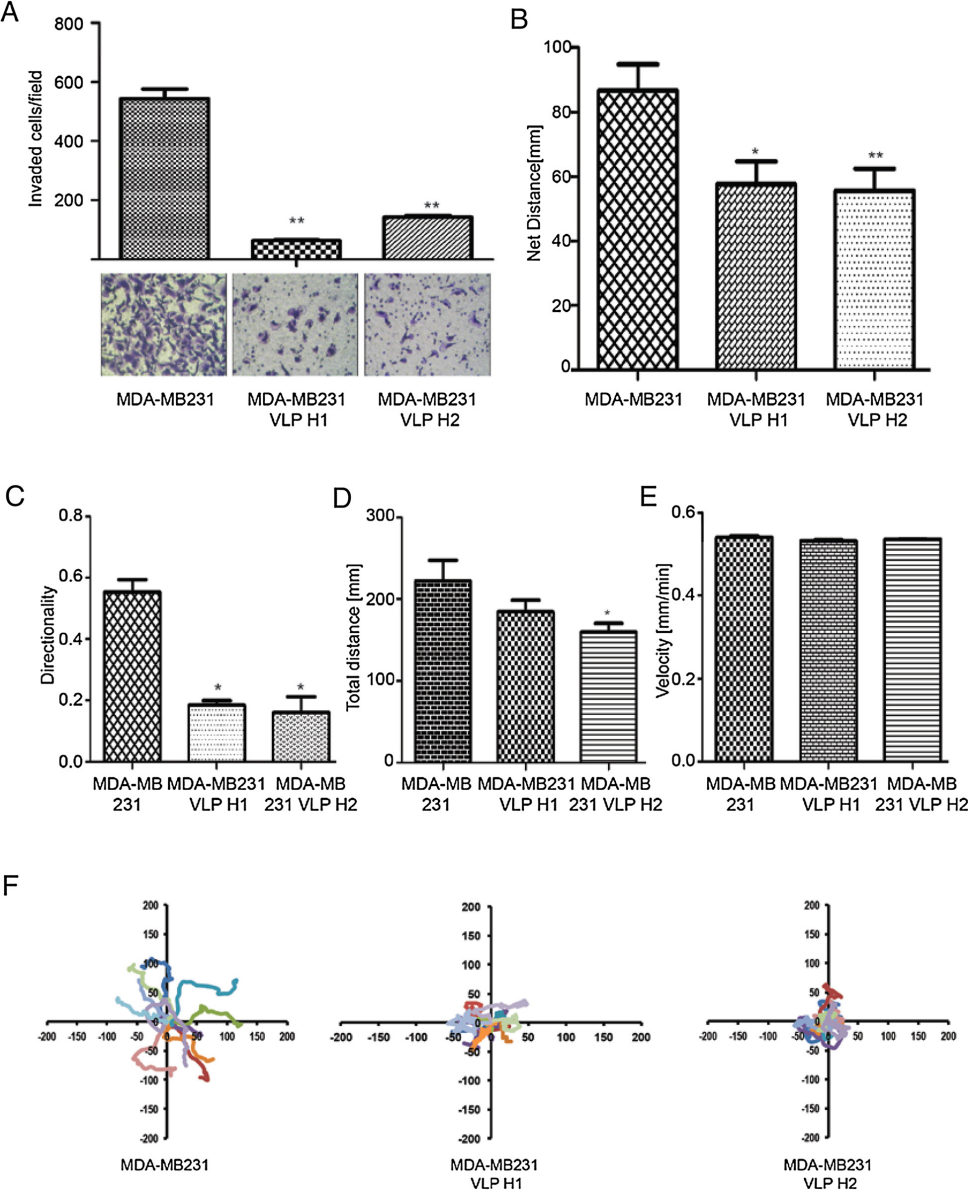
**VLP H1 and VLP H2 inhibit breast cancer cell migration and invasion. (A)** VLP H1 and VLP H2 inhibited the invasion of MDA-MB-231 cells. Data are presented as mean ± SEM, *n* = 5. Ctrl vs VLP H1; Ctrl vs VLP H2, *p* < 0.01. **(B)** Statistic results of net distance of the cells that treated with PBS, 10 μM VLP H1 or VLP H2. **(C)** Statistic results of directionality of the cells that treated with PBS, 10 μM VLP H1 or VLP H2. **(D)** Statistic results of total distance of the cells that treated with PBS, 10 μM VLP H1 or VLP H2. **(E)** Statistic results of velocity of the cells that treated with PBS, 10 μM VLP H1 or VLP H2. The data are expressed as mean ± SEM of more than 60 cells from at least three independent experiments. Single asterisk (*) denotes *P* < 0.05 and double asterisk (**) *P* < 0.01 compared to control. **(F)** Migration tracks of 10 MDA-MB-231 cells that treated with PBS, 10 μM VLP H1 or VLP H2.

IFN-α has an established role in cancer therapy in some cancer types [19-21]. We set out to examine the role of VLP H1 and VLP H2 in breast cancer cell migration and invasion. MDA-MB-231 cells were plated on glass-bottomed dishes coated with 5 μg/ml fibronectin; we then add 10 μM purified VLP H1, VLP H2, or PBS (as control) for 2 h. The migration was determined using time-lapse cell migration assays. VLP H1 and VLP H2 significantly reduced the total distance and directionality of cell migration and strongly inhibited the net distance of cell migration (Figure 3B,C,D,E,F).

To delineate whether VLP H1 and VLP H2 regulate the invasion of breast cancer cells, MDA-MB-231 cells were treated with 10 μM purified VLP H1, VLP H2, or PBS (as control). The invasion of these cells was measured by examining the functional capacities of the cells penetrating through transwell filters coated with 0.35 mg/ml Matrigel. VLP H1 and VLP H2 inhibited the invasion of MDA-MB-231 cells (Figure 3A).

### VLP H1 and VLP H2 inhibit tumor growth in animals

**Figure 4:**
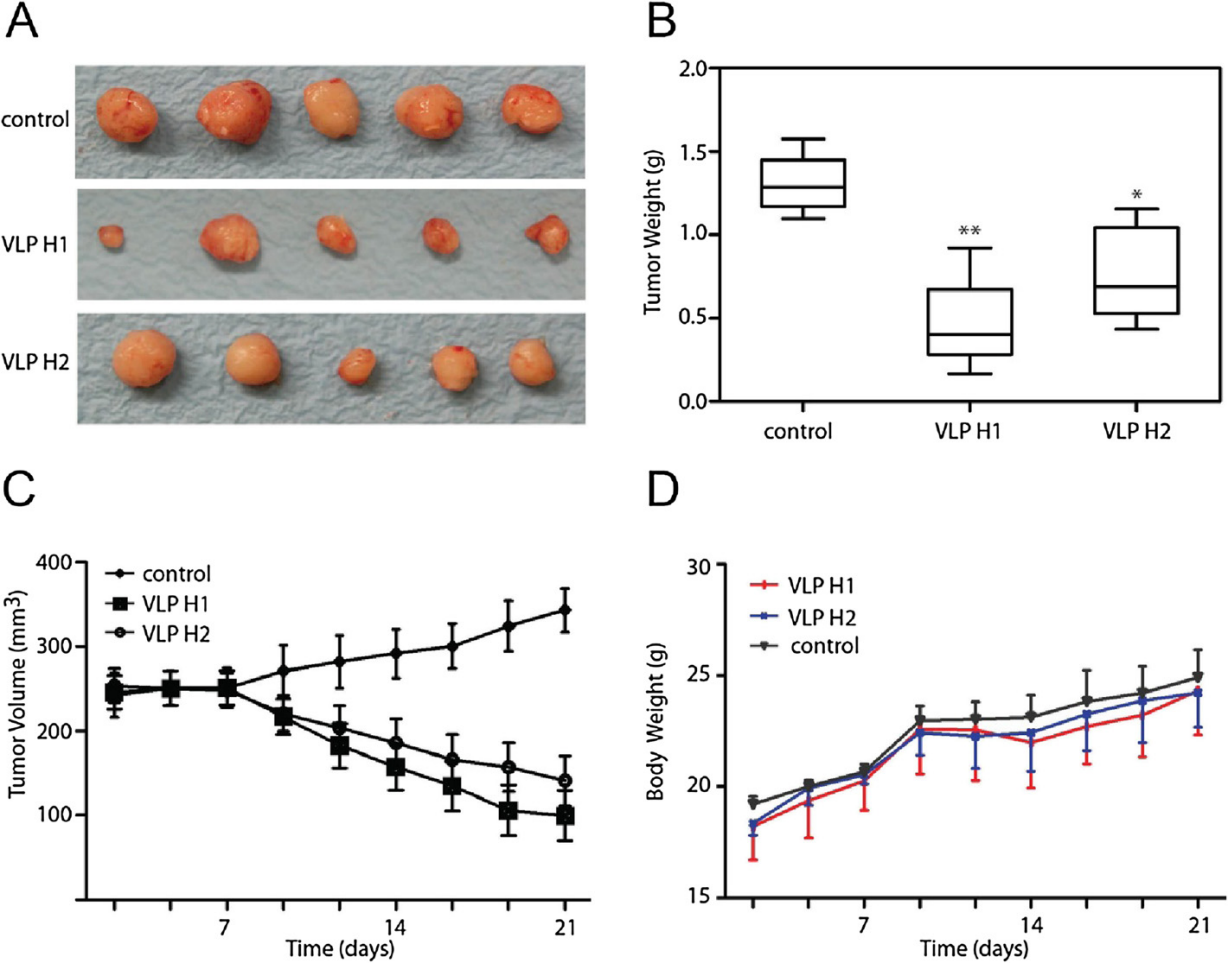
**VLP H1 and VLP H2 suppressed tumor growth in a xenograft model of human breast cancer.** Female nude mice (5 to 6 weeks old) were injected subcutaneously with 1 × 10^6^ MDA-MB231 breast cancer cells into the left and right mammary glands of each animal. Tumor size was measured daily or every other day with calipers, and tumor volumes were calculated using the formula: Volume = (width)^2^ × length/2. After the tumors had established, mice were treated with 10 mg/kg of VLP H1 or VLP H2 (6 days per week) by intraperitoneal injection for 3 weeks. VLP H1 and VLP H2 inhibited tumor growth **(A)**, reduced mouse weight **(B)**, and tumor volumes **(C)** but did not decrease mouse body weights **(D)**.

To evaluate VLP H1 and VLP H2 therapeutic potential, we determined whether VLP H1 and VLP H2 inhibit MDA-MB231 tumor xenograft growth in nude mice. MDA-MB231 cells were implanted in nude mice. After tumors had established, mice were treated with 10 mg/kg of VLP H1 or VLP H2 (6 days per week) by intraperitoneal injection for 3 weeks. VLP H1 and VLP H2 inhibited tumor growth, resulting in significantly reduced tumor volumes (Figure 4C). Indeed, the tumors in VLP H1- and VLP H2-treated mice were significantly smaller (Figure 4A), and 10 mg/kg of VLP H1 and VLP H2 decreased the tumor mass by 64.58% and 41.36%, respectively (Figure 4B). Interestingly, VLP H1 and VLP H2 did not decrease mouse body weights (Figure 4D) - a result consistent with the notion that VLP H1 and VLP H2 preferably target tumor cells and thus exhibited little toxicity to the animals. Taken together, we demonstrated that VLP H1 and VLP H2 inhibited tumor growth *in vivo*.

## Discussion

VLPs are multisubunit self-assembly competent protein structures with identical or highly related overall structure to their corresponding native viruses [22]. The term ‘VLP’ has been used to describe a number of biological objects. Uncharacterized structures with viral morphology that are found in biological samples can include empty structures of viral origin that are not composed of nucleic acids, infectious viruses with chemically or genetically introduced structure modifications and noninfectious, self-assembled gene products resulting from the cloning and expression of viral structural genes in heterologous host systems.

HCV is a member of the Flaviviridae family. Its 9.6-kb RNA genome carries a long open reading frame. This frame is co- and post-translationally cleaved by cellular and viral proteases [23] into structural proteins (core, E1, and E2) and nonstructural proteins (p7, NS2, NS3, NS4A, NS4B, NS5A, and NS5B). Core, E1, and E2, the structural proteins, constitute the major viral components of the viral particles, while the nonstructural proteins are required at multiple levels of the virus life cycle, including viral RNA replication [24] and infectious-particle assembly [25]. The single open reading frame is located between two untranslated regions (UTRs), the 5’ UTR and the 3’ UTR, which contain RNA sequences essential for RNA translation and replication, respectively [26-28]. Falcon et al*.* observed the presence of enveloped VLPs with an average diameter of 65 nm in the cytoplasm and inside cytoplasmic vesicles in HCV-infected patient liver tissue. Smaller enveloped VLPs with diameters ranging from 30 to 55 nm were also localized in the cytoplasm of hepatocytes. All of these VLPs were clearly composed of an inner electron-dense core-like particle surrounded by an envelope. In addition, large numbers of unenveloped VLPs resembling nucleocapsid-like structures of 30 nm in diameter were detected mainly in the cytoplasm and also in the ER membranes [29]. Similarly, Chua et al. constructed HCV virus-like particles using a recombinant adenovirus containing encoding the HCV structural proteins (core, E1, and E2) of HCV 77H, genotype 1a [30].

The baculovirus/insect cell system has been used extensively for the production of VLPs to study viral assembly processes in the absence of infectious viruses, produce antigens for immunization and proteins for diagnostic assays and for gene transfer [31-34]. In this study, various fusion proteins of HCV core, peptides RGD (Arg-Gly-Asp), and IFN-α2a fragments (His-H1, His-H2, His-H3, and His-H4) were successfully expressed via the baculovirus expression system and purified by Ni-NTA agarose. Transcriptional and translational analysis results show that transcriptional levels and expression levels of vAcH1 and vAcH2 are higher than the vAcH3 and vAcH4. His-H1, His-H2, His-H3, and His-H4 all can specifically bind with MDA-MB231. The binding activity of His-H1 and His-H2 is stronger than His-H3 and His-H4 (Figure 1E). The binding activity of His-H1 and His-H2 on MDA-MB231 increased with protein concentration (from 0.5 to 10 μM).

At the same time, HCV core, peptide RGD, and IFN-α2a fragments were expressed by baculovirus expression system and assembled into VLPs. Western blotting results and electron microscopy demonstrate that HCV core, peptides RGD, and IFN-α2a fusion proteins can form 30- to 40-nm-diameter VLPs (Figure 1E,F). Previous experiments showed that IFN provides resistance to virus infection inhibits tumor cell growth and affects the immune function. Our migration and invasion data indicated that VLP H1 and VLP H2 (including IFN-α2a fragments) significantly inhibit MDA-MB231 cells migration and invasion (Figure 3C,D,E,F,G,H). At the same time, *in vivo* studies showed that VLP H1 and VLP H2 inhibit tumor growth in animals (Figure 4).

## Conclusions

In summary, HCV core, RGD (Arg-Gly-Asp), and IFN-α2a fusion proteins can specifically bind tumor cells and self-assemble into 30- to 40-nm-diameter virus-like particles. This interaction can significantly inhibit migration and invasion of MDA-MB231 cells and tumor growth in animals. These results will provide theoretical and experimental basis for the establishment of safe and effective tumor-targeted drug delivery systems and the clinical application of nano-drugs.

## Competing interests

The authors declare that they have no competing interests.

## Authors’ contributions

XL and JXL conceived and designed the experiments. XL, XHX, and AHJ performed the experiments. XL, QYJ, HBZ, and SK analyzed the data. JMG and YLL contributed the materials and analysis tools. XL, JXL, and JMG wrote the manuscript. All authors read and approved the final manuscript.
